# Responding to COVID-19: how an academic infectious diseases division mobilized in Singapore

**DOI:** 10.1186/s12916-020-01641-7

**Published:** 2020-06-08

**Authors:** Sophia Archuleta, Gail Cross, Jyoti Somani, Lionel Lum, Amelia Santosa, Rawan A. Alagha, David M. Allen, Alicia Ang, Darius Beh, Louis Chai, Si Min Chan, See Ming Lim, Dariusz P. Olszyna, Catherine Ong, Jolene Oon, Brenda M. A. Salada, Nares Smitasin, Louisa Sun, Paul A. Tambyah, Sai Meng Tham, Gabriel Yan, Chen Hui Yee, Yock Young Dan, Roland Jureen, Nancy Tee, Malcolm Mahadevan, Ying Wei Yau, Swee Chye Quek, Eugene H. Liu, Clara Sin, Natasha Bagdasarian, Dale A. Fisher

**Affiliations:** 1 Division of Infectious Diseases, Department of Medicine, National University Hospital, National University Health System, 1E Kent Ridge Road, NUHS Tower Block, Level 10, Singapore, 119228 Singapore; 2 Yong Loo Lin School of Medicine, National University of Singapore, Singapore, Singapore; 3 Division of Rheumatology, Department of Medicine, National University Hospital, National University Health System, Singapore, Singapore; 4 Department of Pediatrics, Khoo Teck Puat - National University Children’s Medical Institute, National University Hospital, National University Health System, Singapore, Singapore; 5 Division of Gastroenterology and Hepatology, Department of Medicine, National University Hospital, National University Health System, Singapore, Singapore; 6 Department of Laboratory Medicine, National University Hospital, National University Health System, Singapore, Singapore; 7 Department of Pathology, National University of Singapore, Singapore, Singapore; 8 National Public Health Laboratory, National Centre for Infectious Diseases, Singapore, Singapore; 9 Emergency Medicine Department, National University Hospital, National University Health System, Singapore, Singapore; 10 Department of Anesthesia, National University Hospital, National University Health System, Singapore, Singapore; 11 Group Operations, National University Hospital, National University Health System, Singapore, Singapore

**Keywords:** COVID-19, Pandemic response, Academic infectious diseases

## Abstract

**Background:**

On January 30, COVID-19 was declared a Public Health Emergency of International Concern—a week after Singapore’s first imported case and 5 days before local transmission. The National University Hospital (NUH) is Singapore’s third largest hospital with 1200 beds, heavy clinical workloads, and major roles in research and teaching.

**Main body:**

With memories of SARS still vivid, there was an urgent requirement for the NUH Division of Infectious Diseases to adapt—undergoing major reorganization to face rapidly changing priorities while ensuring usual essential services and standards. Leveraging on individual strengths, our division mobilized to meet the demands of COVID-19 while engaging in high-level coordination, strategy, and advocacy. We present our experience of the 60 days since the nation’s first case. During this time, our hospital has managed 3030 suspect cases, including 1300 inpatients, 37 confirmed cases, and overseen 4384 samples tested for COVID-19.

**Conclusion:**

Complex hospital adaptations were supported by an unprecedented number of workflows and coordination channels essential to safe and effective operations. The actions we describe, aligned with international recommendations and emerging evidence-based best practices, may serve as a framework for other divisions and institutions facing the spread of COVID-19 globally.

## Background

No part of the world will be spared from coronavirus disease 2019 (COVID-19), and every part of every health system needs to adapt. Infectious diseases (ID) physicians and divisions are no longer able to perform many of their usual clinical duties, nor undertake teaching and research as they have always done. There is an urgency to innovate in order to sustainably and safely provide all these essential services while stepping up to and confronting what for many is their raison d’etre. That is the challenge of responding to an emerging infectious disease, keeping pace with the dynamic developments of a pandemic, while designing and refining a seemingly endless number of workflows and policies in partnership with hospital leadership and others beyond the hospital.

This narrative is intended as a guide for ID physicians to help that adaption and to understand the expectations and needs that evolve. The strategy in Singapore, after the initial attempt at preventing local transmission, has been to prevent as many cases as possible and flatten the epidemic curve maximally [1]. Natural herd immunity is not a credible aim unless serological studies reveal unexpectedly high levels of asymptomatic transmission as in Iceland [2]. The endpoint of the outbreak must be an effective antiviral treatment or a vaccine [3]. In the meantime, lives are saved by a containment strategy with early case identification, isolation, and quarantine of close contacts, as well as having adequate hospital surge capacity to deal with spikes. This must be achieved while maintaining a high standard of care for those with COVID-19 as well ensuring our patients with non-COVID-19-related disease receive their usual care. We hope the actions we describe, aligned with international recommendations and emerging evidence-based best practices, may serve as a framework for other ID divisions facing the spread of COVID-19 globally [4].

Our colleagues in many countries may not have had the opportunity to prepare and adapt as we have in Singapore, which is a technologically advanced high-income country with a young highly mobile population. Lockdown is effective but not a permanent solution to contain the spread. Severe restriction of movements and activities will slow transmission and allow an overwhelmed health system to recover albeit slowly. Lockdown should be seen as a time to put in place the measures necessary to prevent a return to uncontrolled spread and, whether a country is early in its phase or overwhelmed, the following viewpoint may nevertheless apply.

## Bracing for COVID-19—division strategy and leadership

On December 31, the World Health Organization (WHO) was informed of a likely novel respiratory pathogen in Wuhan, Hubei, China [5]. The following day, the Emergency Medicine Department (EMD) of the National University Hospital (NUH) began to screen for respiratory illness in returned travelers from Wuhan. As Singapore’s third largest hospital with 1200 beds, NUH serves as the referral center for the nation’s western region [6]. As an academic medical center, it is the primary teaching hospital for the Yong Loo Lin School of Medicine of the National University of Singapore and houses a full complement of tertiary and quaternary care services [7]. These services include those usually provided by a busy academic ID division with sixteen faculty members and four fellows in training [8].

Appreciating the strong economic, cultural, and familial ties between Singapore and China, Singapore anticipated early cases—with potential amplification by travel related to the Lunar New Year beginning on January 25 [9]. Informed by the experience from the severe acute respiratory syndrome (SARS) outbreak in 2003 [10] and tapping on ID expertise, hospital leadership met on January 5 to activate its emergency planning operations and command center. The ID division itself reorganized its operational structure to better meet the anticipated needs. Beyond the immediate leadership of the division, individuals were assigned critical leadership roles and empowered to act independently within the important communication paradigm to enable consistency and coordination (Table 1).

**Table 1:** Division of infectious diseases roles and key responsibilities in the COVID-19 response

**Role**	**Key responsibilities**
Division chief	Provide leadership for the division pandemic response and overall strategy
	Reorganize existing roles, manpower, and other division resources to ensure leadership and business continuity
	Ensure hospital- and national-level advocacy, feedback, and coordination
Clinical director	Oversee divisional rosters and manages clinical team to ensure sustainability
	Develop clinical workflows and protocols in partnership with key stakeholders
	Coordinate with major stakeholders within the department of medicine and hospital (i.e., emergency department, ambulatory services)
Deputy clinical director	Job-share with clinical director to ensure continuity and adequate downtime
	Coordinate with select stakeholders that care for patient populations requiring distinct workflows/protocols (i.e., transplant, hematology-oncology)
IPC director	Lead multidisciplinary IPC efforts at hospital level with national-level advocacy and coordination
	Oversee IPC protocols including personal protective equipment guidance to protect against nosocomial transmission
	Advocate and plan for enhanced screening, isolation, and cohort capacity
ID-IPC liaison	Liaise with key stakeholders requiring enhanced IPC input (i.e., anesthesiology department, intensive care units)
	Coordinate with infection control nurses to audit IPC practices on pandemic wards, operating room workflows
	Partner with occupational health clinic to develop protocols for screening of exposed or unwell staff
Hospital epidemiology director	Oversee hospital contact tracing for confirmed cases, to ensure no IPC breaches and no staff, patients, or visitors exposed
	Synthesize and report data nationally and to hospital leadership
	Manage epidemiology unit and plan for surge manpower
Pandemic team clinicians	Oversee patient care and manage medical teams on wards caring for COVID-19 patients
	Embed in pandemic teams as a COVID-19 resource and review all screened suspect cases
Non-pandemic team clinicians	Ensure continuity of division non-COVID-19 clinical inpatient and outpatient services
	Cross-cover some of the duties of pandemic team clinicians
Research director	Coordinate and prioritize research with clinicians and university basic science departments
	Update literature reviews and summaries of emerging treatment and other COVID-19-related evidence
Fellowship program director	Ensure safety, well-being ,and optimal education-service balance for ID trainees
	Adjust teaching activities to adapt to pandemic response phase and maximize learning opportunities
Media liaison	Coordinate responses to media and public education requests

Suggested roles reflect our experience and may be shared, combined, or contextualized to ensure optimal coverage of key responsibilities

*Abbreviations*: *ID* infectious diseases, *IPC* infection prevention and control

Our strategy, in line with national direction, focused on enhanced screening, surveillance, and isolation of suspected cases [11], as well as ensuring the required resources and manpower were in place both for current needs and stepwise scale-up. We began daily leadership team meetings to review division deployment and operations and to coordinate with hospital stakeholders. An important early realization was that COVID-19-related operations had to be separated from others. This was done primarily through delegation of non-ID clinical work to non-ID faculty within the department of medicine who graciously stepped up to do this, as well as scaling down of routine outpatient consultations, adopting telehealth and home medication delivery. The leadership team needed to be protected from regular duties in order to focus solely and deliver on the pandemic response expectations for which timely response was paramount.

As daily tracking of isolation room capacity and personal protective equipment (PPE) was initiated at the hospital level and plans made for when the state of alert in Singapore was formally escalated, we drafted the first screening and isolation workflows, created PPE guidelines along with holding refresher PPE trainings and risk-stratifying the use of aerosol-generating procedures. We reviewed the early strategy and workflows, discussed the teaching and research activities that should be prioritized, and made a commitment that the division would be on the frontline of the COVID-19 response in whatever capacity was ultimately required. It was time to demonstrate the value of our chosen specialty, and as a division, we chose to make this our moment to shine.

## Ramping up—diagnostic testing, surge isolation capacity, and manpower

On January 23, Singapore reported its first imported COVID-19 case [12]. During this period, a trickle of patients with a history of travel and acute respiratory illness (ARI), none particularly suspicious for COVID-19, had been isolated on admission to NUH and de-isolated based on SARS-CoV-2 PCR testing developed by Singapore’s National Public Health Laboratory (NPHL). The NPHL-based laboratory-developed test was a real-time PCR targeting the N and ORF1ab genes [13]. This test was validated and rolled out at NUH on February 3, and thereafter, we had independent capacity to test all necessary cases in house.

**Fig. 1:**
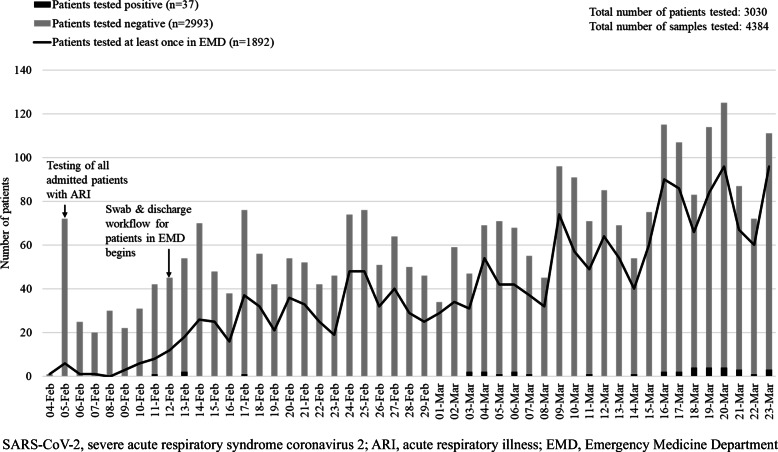
Laboratory testing for SARS-CoV-2 at the National University Hospital

On February 4, when Singapore reported its first local cluster [14], NUH was therefore ready for two important actions. First, this permitted us to conduct once-off testing for SARS-CoV-2 of all current admitted patients with ARI. No cases of COVID-19 were found among the 66 swabs performed that day. This exercise ensured that no undetected cases were on our wards. It also tested the lab’s capacity (which quickly ramped up from 120 to 1600 tests per day) and turnaround time. Second, we implemented enhanced surveillance. From February 5 onwards, all patients admitted to our hospital with evidence of ARI, regardless of travel history, were tested for COVID-19 (Fig. 1). Pending results, patients were isolated in single rooms, rather than cohorted in available open cubicles, to prevent patient-to-patient transmission.

**Fig. 2:**
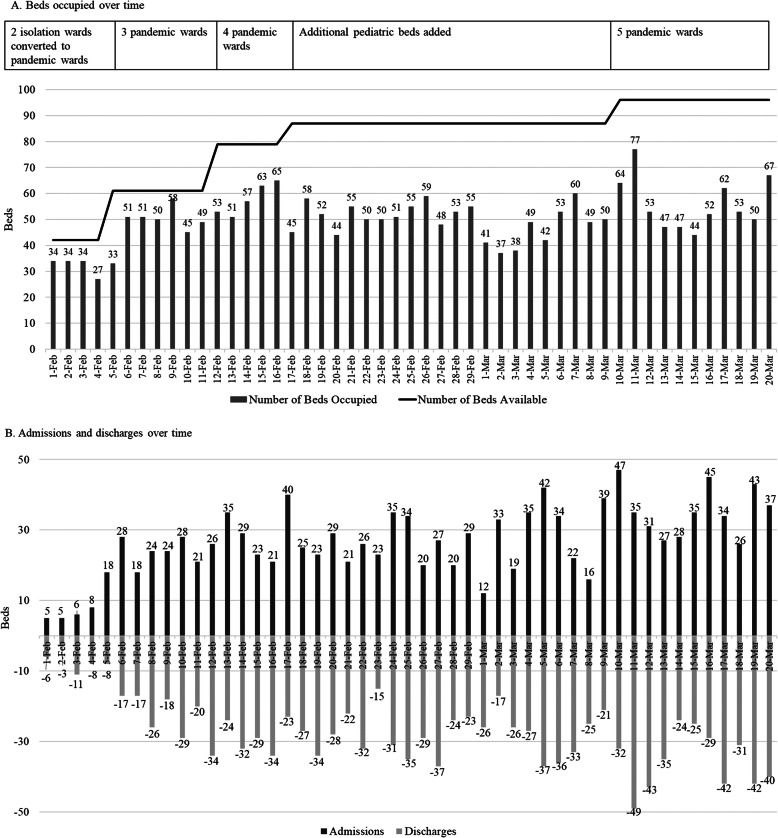
**a** Incremental scale-up and occupancy of isolation capacity. **b** Pandemic ward admissions and discharges over time at the National University Hospital

To accommodate the surge in isolation room requirements, our hospital’s two dedicated isolation wards, with a peacetime capacity of 42 negative pressure rooms, were decanted of existing patients (except for those requiring airborne isolation for other infections such as pulmonary tuberculosis or disseminated zoster) and converted into “pandemic wards.” An additional 3 wards with single rooms and dedicated bathrooms (1 with negative pressure and 2 with neutral pressure) in the private wing of the hospital were decanted and activated as pandemic wards in stages over the following 5 weeks to meet the incremental surge requirements (Fig. 2).

Handling the expected surge of suspected and confirmed COVID-19 patients, while maintaining the department’s usual clinical services, was a major challenge. Our team segregated into 2 teams to minimize cross-exposure should a breech occur—a lesson learned from SARS when transmission in our hospital occurred in a non-SARS ward among general medical patients [15]. This resulted in a complex “pandemic roster” of up to 6 pandemic ward teams, which could be activated in stages and separated from the main roster. The first pandemic team was staffed by the ID division, with the remaining teams staffed by other medical specialties and augmented by an embedded ID specialist on each ward. The embedded ID attending helped incoming non-ID faculty and residents build familiarity with COVID-19-related care and workflows, approved all de-isolation decisions (typically clinical improvement plus 2 negative PCRs at least 24 h apart), reinforced appropriate PPE and other infection prevention practices, and served as a strategic link between the ID leadership team and inpatient frontlines.

Such a significant redeployment of manpower was made possible by a decision to defer non-urgent outpatient visits and routine follow-ups to all medical specialties—ultimately leading to a reduction across the department of medicine’s ambulatory workload of 50–70%. Our workforce was further freed up by the temporary cancelation of medical student teaching on February 7 and other non-clinical activities. To date, the pandemic teams have admitted more than 1300 patients with suspected or confirmed COVID-19 into the designated pandemic wards. They have been responsible not only for identifying and managing those with confirmed infection but, equally importantly, for maintaining flow through the pandemic wards by safely and efficiently de-isolating those without COVID-19. Only select patient groups with ARI, for example stem cell transplant patients, are cared for in consultation with ID in isolation beds within their units, ensuring they receive the specialist nursing care they require.

The clinical leaders meet daily to discuss and respond to hospital-wide concerns, troubleshoot problems, and provide a forum for feedback from the medical teams on the ground. We receive detailed isolation bed occupancy statistics and act quickly in response to admission spikes to avoid a bed-block situation. To maintain outflow from the pandemic wards, teams were granted priority and “disposition rights” to transfer with appropriate handover their de-isolated patients to any medical or surgical team best suited to the further care of the patients. Timely results became critical as isolation bed occupancy rates spiked. With strong support from the lab, ID advocated for in-house SARS-CoV-2 PCR testing to be scaled up from 2 to 3 runs a day to eventually 5 runs a day (with capacity for 300 tests per day). Starting on March 2, testing was conducted using a commercial kit (A*STAR Fortitude Kit 2.0, SARS CoV-2 RT-PCR Test, Agency for Science, Technology and Research, Singapore) with assays running from 7 am to midnight and a turnaround time of 6 h. Well patients were readied for anytime of the day or night discharges, which nurses were empowered to execute after checking for negative results.

To cope with increased flow into the pandemic wards, we advocated with EMD for extended screening capacity as well as a “swab-and-go” service for well patients with ARI to be screened and discharged home with instructions to self-isolate pending results. The service included patients being informed of negative results and instructions for further care via secure automated messaging, while positive results were notified by an ID physician, who arranged direct admission to one of the pandemic wards. As of March 23, 4384 SARS-CoV-2 tests have been conducted at our institution, on 3030 patients, with 37 (1.2%) confirmed COVID-19 cases. Of the total, 1982 tests (45.2%) have been performed in the EMD or outpatient setting. Of the 37 positive patients, 15 were tested under our EMD “swab-and-go” workflow.

On March 21, when our isolation bed occupancy rate reached a predetermined trigger point of 80% plus 10 inpatient COVID-19 cases, we opened a dedicated 32-bed open ward with 6-bed cubicles to cohort all COVID-19 patients, manned by an ID physician-led 6th pandemic team. Prior to opening the cohort ward, and with non-COVID-19 inpatients still present, a 4-h-long simulation was held, with staff wearing full PPE under observation, understanding the actions and movements necessary to maintain excellent infection prevention practices. This action reserved our isolation beds primarily for suspect cases that would be transferred to the cohort ward if confirmed positive. Infection prevention practices on this cohort ward remain a priority with an embedded infection prevention nurse who performs audits on a daily basis and provides feedback to the staff on the ground as well as to the hospital leadership.

## An outbreak of workflows, meetings, and communications

An unprecedented number of workflows were developed from scratch, existing hospital pandemic drawer plans, or by adapting guidance and shared practices by Singapore’s National Centre for Infectious Diseases [16, 17] and Ministry of Health [18]. Creating, updating, and coordinating these in a necessarily collaborative way required a dedicated effort. By February 10, the workflows and guidelines developed were collated into a hospital-wide COVID-19 Emergency Preparedness and Response Plan, which was disseminated to all medical personnel at NUH, hosted on our intranet and continues to be built upon (Table 2).

**Table 2:** Workflows created for COVID-19 emergency preparedness and response plan

**Workflows by sector**	**Key elements**
**Adult and children’s emergency department**	
Assessment of patients with ARI	Screening, clinical assessment, and risk stratification of COVID-19 suspects for admission versus “swab-and-go”
Discharge of well patients with ARI (“swab-and-go”)	Patient discharge criteria and advice with instructions for self-isolation, process for result notification, and return advice
Notification and follow-up of patients “swab-and-go” results	Notification of SARS-CoV-2 test result—automated messaging of negatives, phone notification by ID, and direct admission of positives
Admission of family clusters with ARI	Coordinated workflow with medicine and pediatrics, including bed assignment for parents and children with suspect/confirmed COVID-19 to stay together
**Ambulatory setting**	
Assessment of outpatients with ARI	Screening, clinical assessment, and risk stratification of COVID-19 suspects for referral to emergency department, direct admission to isolation or “swab-and-go” with special attention to routes dedicated for patient movement
Screening of visitors to ambulatory centers	Self-declaration of symptoms and travel history, and thermal scanning of all visitors (and patients) with strict limit of 1 visitor per patient
**Inpatient setting**	
Admission to pandemic wards	Appropriate placement of suspect and confirmed cases based on risk and incremental surge isolation capacity to minimize nosocomial transmission risk and rationalize use of isolation rooms
De-isolation of suspect and confirmed COVID-19 patients	Appropriate clinical assessment, as well as testing strategy (frequency and type of specimens) in relation to level of clinical and epidemiological suspicion before de-isolating patients, as well as discharging them home or to community isolation facilities
Assessment of inpatients on non-pandemic wards with ARI	Clinical assessment and risk stratification to determine need for testing and transfer to pandemic ward
Admission and management of suspect and confirmed COVID-19 cases in select patient populations	Individualized workflows for immunocompromised hosts, pregnant women, patients requiring surgery or aerosol-generating procedures
Critical care of suspect and confirmed COVID-19 cases	Protocols, including PPE guidance, for patient requiring cardiopulmonary resuscitation, endotracheal intubation, tracheostomy, extracorporeal membrane oxygenation
**Staff safety and management**	
Assessment of staff with ARI with or without known workplace or community COVID-19 exposure	Risk assessment and testing following staff exposure incidents based on PPE worn, procedure performed, duration, and proximity to patient
Management of staff returning from overseas travel	Management of staff under quarantine order or stay-home notice
Staff temperature surveillance	Twice-daily temperature checks and online recording in surveillance system
Guidance on appropriate use of PPE	Guidance on PPE according to clinical area and type of patient contact, including aerosol-generating procedures

*Abbreviations*: *ARI* acute respiratory illness, *SARS-CoV-2* severe acute respiratory syndrome coronavirus 2, *PPE* personal protective equipment

Communicating the COVID-19 response strategy and workflows became a key component of our regular operations. Once acceptance of any workflow or policy was agreed upon by stakeholders, channels of communication, including “town halls,” while large gathering were still permitted; huddles with key individuals; and WhatsApp® group chats with the pandemic teams, epidemiology teams, and others, permitted rapid dissemination of information and feedback. While public messaging applications are user-friendly, a hospital-approved, Health Insurance Portability and Accountability Act compliant messaging service is vital for patient-specific information. Such a dedicated service was utilized to rapidly share newly positive patients by the lab with the ID clinical and epidemiology teams; to discuss specialized pathways to be activated, including special routes of transfer; or to expedite laboratory testing for specific patients. Video and teleconferencing applications have also played a vital role in keeping lines of communication open between frontline staff and key stakeholders, especially when social distancing is encouraged, and can bring together stakeholders across institutions, allowing for close alignment of hospital policy and procedures across a health system.

Another key communications platform was the early institution of a 24-h telephone hotline manned by an ID attending, to ensure patients were right-sited into and out of pandemic wards, provide reassurance in the interpretation of complicated workflows, and allay anxieties which ranged from the management of unwell patients to feedback on infection control lapses. The “Coronavirus Consultant Hotline” remains an indispensable feature of our communications where not every situation can be captured via workflow. Since its inception on February 3, it has received an average of 45 calls per day.

Beyond technological communications, however, we found urgent face-to-face meetings or briefings were required to defuse situations involving heightened sensitivities. Such interactions were needed with departments facing staff exposures or concerns of higher risk by virtue of their work. Examples from our experience include anesthesiologists, pulmonary function lab staff, pathologists, and porters. Regardless of method, messaging needs to be consistent, accurate, and from someone trusted. It ideally should be delivered by a selected few whether it be to the media, hospital management, clinicians, or others. The fastest way to lose credibility (which impacts the capacity to succeed) is to provide poor or inaccurate information. Furthermore, contextualizing the information provided and acting on feedback is critical in communications.

## Redefining our academic mission

The impact of COVID-19 on the academic priorities of our division cannot be overstated. As an academic division with a 3-year fellowship program, and active bench and clinical research programs, reorganization of this portfolio was urgently needed. An early decision was made to protect some of the time of the division’s three clinician-scientists to focus on COVID-19-related studies of diagnostic and treatment modalities. We committed as a group that while we might offer antiviral treatment on a compassionate basis to our patients with COVID-19, our goal was to ultimately participate in international, rigorously conducted randomized clinical trials. All agreed that despite exigencies of clinical service, duty to our patients included generating evidence that would meaningfully contribute to the field. This approach has so far led to five grants won, initiation of a specimen biobank and associated BSL2+ facility, four prospective cohort studies, and two interventional therapeutics RCTs.

Our division also committed to fully leverage on the unique learning opportunities afforded to our four ID fellows by the COVID-19 response. The division’s chief and clinical director worked closely with the fellowship program’s director and “chief fellow” on an incremental clinical surge roster for the fellows, balancing involvement on the pandemic teams with other required clinical experiences. Similar to the actions taken for ID faculty, this was further facilitated by a reduction of fellows’ routine outpatient workload. Regular structured didactics were replaced by just-in-time learning on topics ranging from how to keep abreast with the evidence being rapidly generated to basic epidemiology principles. COVID-19 also afforded rich case studies for faculty to teach professionalism and medical ethics in an outbreak setting such as public health exceptions to patient confidentiality, mandated isolation, and quarantine orders. As face-to-face group teaching was suspended to minimize transmission risk, we adapted to use teleconferencing and virtual chat groups for learning. Training was also rapidly implemented to mitigate risk of COVID-19 to fellows such as personal protective equipment refreshers, regular updates on hospital workflows, and protocols on managing suspected and confirmed cases. A concerted effort was made to extend standing invitations to our future ID specialists to the hospital’s high-level preparedness and response meetings.

## Conclusions

Every city of every country is at a different phase of their own outbreak. Singapore has managed its outbreak response with a strong commitment to containing the virus to minimize social disruption. All suspect cases are admitted to hospital and isolated. Others are tested and allowed home with strict instructions to self-isolate until results are communicated to them, usually within hours. All confirmed cases remain isolated in hospital (or more recently, community isolation facilities) [19]. Close contacts and overseas arrivals are in strict quarantine. This is the practice in at least five countries, none of which is in a community transmission phase.

Overwhelmed countries with community transmission and under some degree of lockdown may not yet be able to implement much of what we have presented. However, as transmission is brought under control, there will be a need to adapt hospital workflows in a way similar to those described. The ID divisions will be called on to show leadership in every aspect of hospital function. There needs to be adaptability, agility, and strong leadership within the division. Each individual needs to understand their role, and do it well and fast. Everyone needs a colleague backup as no one can be indispensable. Decisions and policies need a blend of science and empathy, while communications and messaging are paramount. ID divisions globally are being tested, and this is our moment to shine.

## Data Availability

Not applicable

## Abbreviations

ARI: Acute respiratory illness
COVID-19: Coronavirus disease 2019
ID: Infectious diseases
EMD: Emergency Medicine Department
NPHL: National Public Health Lab
NUH: National University Hospital
PPE: Personal protective equipment
SARS: Severe acute respiratory syndrome
SARS-CoV-2: Severe acute respiratory syndrome coronavirus 2
WHO: World Health Organization

## References

[1] Anderson RM, Heesterbeek H, Klinkenberg D, Hollingsworth TD (2020). How will country-based mitigation measures influence the course of the COVID-19 epidemic?. Lancet.

[2] Day M (2020). Covid-19: identifying and isolating asymptomatic people helped eliminate virus in Italian village. BMJ.

[3] Wong JEL, Leo YS, Tan CC. COVID-19 in Singapore-current experience: critical global issues that require attention and action. JAMA. 2020;323(13):1243-4. 10.1001/jama.2020.2467 32077901

[4] Adalja AA, Toner E, Inglesby TV. Priorities for the US health community responding to COVID-19. JAMA. 2020;323(14):1343-4. 10.1001/jama.2020.3413 32125355

[5] Wang C, Horby PW, Hayden FG, Gao GF (2020). A novel coronavirus outbreak of global health concern. Lancet.

[6] National University Health System (2019). About NUH: overview.

[7] National University Health System (2019). Education@NUHS.

[8] National University Health System (2020). Our services: infectious diseases.

[9] MOH Steps Up Precautionary Measures In Response To Increase In Cases Of Novel Coronavirus Pneumonia In Wuhan [press release]. January 20 2020.

[10] Singh K, Hsu LY, Villacian JS, Habib A, Fisher D, Tambyah PA (2003). Severe acute respiratory syndrome: lessons from Singapore. Emerg Infect Dis.

[11] Ng Y, Li Z, Chua YX (2020). Evaluation of the effectiveness of surveillance and containment measures for the first 100 patients with COVID-19 in Singapore - January 2-February 29, 2020. MMWR Morb Mortal Wkly Rep.

[12] Confirmed imported case of novel coronavirus infection in singapore; multi-ministry taskforce ramps up precautionary measures [press release]. January 23 2020.

[13] Kam KQ, Yung CF, Cui L (2020). A well infant with coronavirus disease 2019 (COVID-19) with high viral load. Clin Infect Dis.

[14] Pung R, Chiew CJ, Young BE, et al. Investigation of three clusters of COVID-19 in Singapore: implications for surveillance and response measures. Lancet. 2020;395:1039-46. 10.1016/S0140-6736(20)30528-6 PMC7269710 32192580

[15] Fisher DA, Chew MH, Lim YT, Tambyah PA (2003). Preventing local transmission of SARS: lessons from Singapore. Med J Aust.

[16] National Centre for Infectious Diseases S (2020). National Centre for Infectious Diseases.

[17] Tay JY, Lim PL, Marimuthu K, et al. De-isolating COVID-19 Suspect Cases: A Continuing Challenge [publishedonline ahead of print, 2020 Feb 26]. Clin Infect Dis. 2020;ciaa179. 10.1093/cid/ciaa179.

[18] Ministry of Health S (2020). Updates on covid-19 (coronavirus disease 2019) local situation.

[19] 16 more cases discharged; 35 new cases of covid-19 infection confirmed [press release]. March 30 2020.

